# Efficacy and Safety of Intranasal Esketamine in Treatment-Resistant Depression with Comorbid Autism Spectrum Disorder: Three Case Reports

**DOI:** 10.3390/clinpract16030061

**Published:** 2026-03-13

**Authors:** Alessandro Guffanti, Matteo Leonardi, Natascia Brondino, Bernardo Dell’Osso, Vassilis Martiadis, Miriam Olivola

**Affiliations:** 1 Department of Brain and Behavioural Sciences, University of Pavia, 27100 Pavia, Italy; 2 Department of Mental Health and Addiction Services, Azienda Socio-Sanitaria Territoriale di Pavia, 27100 Pavia, Italy; 3 Department of Mental Health, Department of Biomedical and Clinical Sciences Luigi Sacco, University of Milan, 20157 Milan, Italymiriam.olivola@asst-fbf-sacco.it (M.O.); 4 “Aldo Ravelli” Centre for Neurotechnology and Brain Therapeutic, University of Milan, 20143 Milan, Italy; 5 Department of Psychiatry and Behavioural Sciences, Bipolar Disorders Clinic, Stanford University, Stanford, CA 94305, USA; 6 Department of Mental Health, Azienda Sanitaria Locale Napoli 1 Centro, 80145 Naples, Italy

**Keywords:** case report, major depressive disorder, treatment-resistant depression, autism spectrum disorder, esketamine, suicidal ideation, mentalization, social cognition

## Abstract

Introduction: Major depressive disorder (MDD) is a leading cause of disability worldwide and contributes significantly to the global burden of disease. Recent data show an increasing prevalence of treatment-resistant depression (TRD). Patients with autism spectrum disorder (ASD) often exhibit MDD as a comorbidity and it is often resistant to conventional treatments. ASD determines emotional dysregulation and a reduced ability to understand mental states (mentalization). These features can lead to suicidal ideation and/or behavior. Intranasal esketamine may offer a novel therapeutic option for this population. Methods: This case series focuses on the clinical response to intranasal esketamine in patients with autism and TRD; esketamine is approved in Italy as an add-on therapy in TRD, so our case study is based on an in-label treatment. Three young patients (*n* = 3, F/M 2:1, age range 20–25 y) with light to moderate autism (Level 1 or 2) were treated. Esketamine was administered in augmentation with selective serotonin reuptake inhibitors (SSRIs) or serotonin-norepinephrine reuptake inhibitors (SNRIs) in accordance with EMA/AIFA guidelines. A structured follow-up protocol was set to monitor depressive symptoms, social cognition, and mentalization. Follow-up during treatment was maintained for six months, and psychometric evaluations were performed at six time points: baseline (T0), 1 week (T1), 1 month (T2), 2 months (T3), 3 months (T4), and 6 months (T5). Also, subjective quality of life was investigated before and after the observation period. Results: Despite differences in clinical profile, all patients showed good efficacy of esketamine in reducing depressive symptoms: two patients experienced clinical remission at T5 (MADRS < 10), one patient showed partial response (dMADRS = 43.24%). No major side effects were reported. Significant improvements were observed after the first week of treatment (P1: MADRS_T0 = 37, MADRS_T1 = 12; P2: MADRS_T0 = 32, MADRS_T1 = 21; P3: MADRS_T0 = 25, MADRS_T1 = 12). Depressive relapses occurred (e.g., P1, T3–T4), but they were not associated with hospitalizations and/or suicidal attempts. Suicidal ideation, when present, decreased by the end of the follow-up period. Lack of mentalization and in social cognition was noted, with just mild improvements during therapy. Subjective quality of life improved significantly for all patients (P1: 28% at T0, 73% at T5. P2: 25% at T0, 71% at T5. P3: 35% at T0, 80% at T5). Conclusions: Intranasal esketamine showed a favorable efficacy and safety in these three cases of TRD in comorbidity with ASD (at six months: total remission = 66.66%, partial remission = 33.33%, inefficacy = 0%, drop-out = 0, severe adverse events = 0). Besides improvements in depressive symptoms, esketamine was associated with a constant decrease in suicidal thoughts. A case series is unfit to form statistical conclusions; preliminary data warrant further investigation in randomized controlled studies to validate the therapeutic potential of esketamine in this population.

## 1. Introduction

Major depressive disorder (MDD) is a major cause of disability and contributes to suicide deaths worldwide. In 2018, MDD was ranked third in terms of disease burden, and it is predicted to rank first by 2030 [1]. The prevalence of MDD in 2025 is 5.7% among the worldwide population and is more prevalent in women (6.9%) than in men (4.6%) [2]. At the same time, the prevalence of treatment-resistant depression (TRD) is increasing. TRD is defined as a depressive episode that does not improve adequately after almost two pharmacological trials appropriate in dose and duration [3]. Major depression is a very frequent comorbidity, considering both medical and psychiatric conditions [4]. Autism spectrum disorder (ASD) is frequently associated with anxious and/or depressive symptoms, with a reported prevalence of 20% and 9% respectively [5]. Recent evidence from a systematic review indicates that individuals with ASD are at nearly three times greater risk of suicide mortality than neurotypical individuals, particularly in the absence of co-occurring intellectual disability [6]. This finding could be explained by the impairment in mentalization skills. The difficulty in understanding mental states can reduce tolerance to everyday life stressors—i.e., family, work, relationships—and suicidal thoughts can develop as an “escape path”. Resistance to change, rigid thought patterns, and emotional dysregulation are common symptoms in ASD, and they could contribute to an increase in suicidal risk [7,8,9]. Emotional dysregulation was associated with suicidal behaviors, especially in young patients [10]. MDD in ASD represents a serious pathological condition that requires early diagnosis and efficient treatment. ASD patients show lower response to pharmacological treatments due to several factors, such as genetic variability [11], cognitive rigidity, and comorbid conditions. There are no approved pharmacological treatments for the core symptoms of ASD; available medications focus on managing symptoms without targeting the underlying neurobiological mechanisms [12]. A personalized approach can be recommended, combining a targeted therapy with major symptoms and non-pharmacological interventions (behavioral, dietary, environmental) [13]. In our experience, we focused on depressive symptoms in ASD, considering a potential treatment-resistant profile. We administered intranasal esketamine (Spravato^®^) to three young ASD patients who had severe depressive symptoms and SI, not responding to oral antidepressants. Esketamine is approved in Italy as a therapeutic option in TRD used in augmentation with SSRI or SNRI medications [14], so an in-label intervention was carried out. Esketamine is the S-enantiomer of ketamine and a non-selective and non-competitive N-methyl-D-aspartate (NMDA) receptor antagonist. The molecule binds receptors on GABAergic interneurons (inhibitory), causing release of glutamate in synaptic gaps; glutamate via α-amino-3-hydroxy-5-methyl-4-isoxazolepropionic acid (AMPA) receptor determines excitatory stimulation in the cortex and in the limbic system, with an augmented production of neurotrophic factors. The fast antidepressant effects of esketamine were associated with direct stimulation of the mammalian target of rapamycin complex 1 (mTORC1) and the stimulation of a molecular pathway involved in brain-derived neurotrophic factor (BDNF) synthesis [15]. Remodulation involving the glutamate pathway and neurotrophic factors could be potentially useful in autism comorbidity, which has been associated with molecular mechanisms like glutamate-induced excitotoxicity and oxidative stress (e.g., neuroinflammation) [16].

## 2. Methods

### 2.1. Study Design, Setting, and Goal

This study was a small case series with a total of three patients recruited in public mental health services. The design has been selected to share data regarding people with TRD and ASD comorbidity treated with intranasal esketamine. Moreover, a case series offers the possibility of a more detailed description of patients, from demographic features to psychopathological aspects.

Inclusion criteria: patients with ASD and moderate to severe MDD who met the criteria for the TRD, as explained.

Exclusion criteria: patients with active psychotic symptoms and/or severe cardiovascular conditions.

Pharmacological treatment and clinical follow-up were carried out in mental health services of Pavia (P1 and P2) and Milan “Fatebenefratelli-Sacco” (P3). All participants received treatment as outpatients. P2 underwent the first part of the follow-up (T0 and T1) during his inpatient stay in the Psychiatric Ward of Pavia. Esketamine clinics of Pavia and Milan “Fatebenefratelli-Sacco” are specialized in treating TRD patients, according to guidelines in Italy. Both services are characterized by experience in diagnosis and approach to TRD, from pharmacological administration to clinical assistance and follow-up. The following case reports describe the first three patients with TRD and ASD treated in our clinics.

### 2.2. Participants

Three ASD patients were recruited from 2023 to 2025. Clinical and socio-demographical information were summarized in Table 1.

ASD diagnosis was carried out with a multidisciplinary approach, combining neuropsychological tests and the clinical opinion given by expert psychiatrists. Neuropsychological evaluation was carried out with the Autism Diagnostic Observation Scale (ADOS), Social Responsiveness Scale (SRS), and the Wechsler Adult Intelligence Scale (WAIS-IV). Patients enrolled were affected by mild to moderate autism (Level 1 or 2, according to the *Diagnostic and Statistical Manual of Mental Disorders 5, Text Revision*). At baseline, all patients were characterized by resistant depressive symptoms with or without suicidal ideation. P2 and P3 were treated with esketamine for the first time in this protocol. P1 had already undergone a previous trial with esketamine (2021) with partial benefit, interrupted for subjective improvement (see below, “Results—Patient 1”). A preliminary visit was performed to assess the necessity of intervention and patient compliance, providing all information about esketamine and psychometric evaluation.

### 2.3. Intervention

Esketamine treatment was performed in accordance with the EPA/AIFA guidelines, in augmentation with selective serotonin reuptake inhibitors (SSRIs) or serotonin-norepinephrine reuptake inhibitors (SNRIs). All patients received administration twice a week for the first month and weekly for the second month, as specified in the technical data sheet. After this period, a personalized frequency of administration was set depending on the clinical picture: patients with slight improvement or clinical stability maintained two administrations/month, patients with significant improvement shifted to one administration per month. When depressive relapses occurred, the return to weekly administration was considered. None of the three patients showed serious side effects during treatment, and they all received the maximum dosage of esketamine (84 mg per administration, continued for the whole observation period). Once they demonstrated good tolerability to intranasal esketamine, the maximum dosage was prescribed to maximize the probability of response in a TRD; no dose adjustment was required. During treatment, oral pharmacotherapy with an SSRI or SNRI was continued with good compliance (P1: sertraline 200 mg/day, P2: venlafaxine 300 mg/day, P3: escitalopram 20 mg/day). After esketamine administration, patients remained under clinical observation for at least one hour, monitored by clinicians and nurses. More information regarding esketamine pharmacotherapy (e.g., frequency of administration, changes in posology, vital signs) is reported in the Supplementary Material.

### 2.4. Outcomes

The primary outcome was to evaluate clinical response to esketamine, which has been defined as a decrease in MADRS total score of ≥50% from baseline (T0). Secondary outcomes were evaluating changes in social cognition and/or mentalization; assessing the severity of psychache; focusing on suicidal ideation and/or behaviors; and lastly, highlighting patients’ experience in terms of quality of life and subjective condition.

The psychometric assessment was composed of six tests and scales (Table 2), administered by clinicians at six time points.

- Montgomery–Asberg Depression Rating Scale (MADRS): a highly used 10-item questionnaire. MADRS is often selected to monitor depressive symptoms and define the severity of MDD during follow-up; its validity as clinical predictor has been confirmed in TRD [17].
- Psychache Scale (PS): a 13-item scale used to assess the profile of psychological pain. Some questions demand a frequency rating (from “never” to “always”), while other ones demand an agreement rating (from “strongly disagree” to “strongly agree”). Correlation between unbearable psychache and SI in patients affected with MDD was described [18].
- Columbia-Suicide Severity Rating Scale (C-SSRS): a structured test to evaluate suicidal ideation and/or behavior. It is composed of 10 categories, of which the majority call for a binary response yes/no (e.g., suicidal attempts yes/no). When suicidal thoughts and/or behavior were present, the sub-scale, which measures the intensity of ideation (1–5), was reported and considered as a follow-up tool. C-SSRS was used to monitor SI during the observation period in our case reports, but its score is unfit to compare SI in different patients [19].
- Quality of Life Enjoyment and Satisfaction Questionnaire (Q-LES-Q): a subjective investigation about quality of life, which includes different domains (school, work, housework, social relationships, physical state, leisure time, general activities). Every sub-score can be associated with a percentage of satisfaction: the average of these eight percentages defines the global quality of life perceived by patients. Q-LES-Q (58 items) and Q-LES-Q Short Form (16 items) are used as predictors of disability and quality of life in MDD [20].
- Reading the Mind in the Eyes Test (RMET): a specific test which measures the theory of mind and the ability to recognize mental states in other people. It consists of 36 different photographs: for each, the patient should indicate what person in the picture is feeling/thinking. It has been noticed that autistic people underperform on this test as compared to the neurotypical population [21].
- Reflective Functioning Questionnaire 8 items (RFQ-8): a brief, easy-to-administer screening to evaluate mentalization, coming from a 54-item test [22]. It is focused on reflective functioning, intended as the ability to recognize our feelings and our mental states before acting. This test is organized as a Likert-type scale, with responses from “completely agree” (7) to “completely disagree” (1). Higher scores in RFQ-8 show a lower ability in mentalization.

**Table 2:** Assessment tools.

DOMAIN	SCALE	ABBREVIATION	SCORE
Depression	Montgomery–Asberg Depression Rating Scale	MADRS	0–60
Psychological pain	Psychache Scale	PS	13–65
Suicidality	Columbia-Suicide Severity Rating Scale	C-SSRS	If present, assess “Ideation” subscale 1–5
Social Cognition	Reading the Mind in the Eyes Test	RMET	0–36
Mentalization	Reflective Functioning Questionnaire (8 items)	RFQ-8	1–7
Quality of Life	Quality of Life Enjoyment and Satisfaction Questionnaire	Q-LES-Q	0–100%

These tests were administered at T0 (before esketamine), T1 (1 week of treatment), T2 (1 month), T3 (2 months), T4 (3 months), and T5 (6 months). In our study design, blind rating was not feasible due to logistic limitation (clinicians who administered the tests were the same ones responsible for pharmacological treatment). The window period for tests and administrations was ±5 days for each time point. Q-LES-Q was evaluated only at T0 and T5: this choice was motivated by the nature of the questionnaire, which investigates domains with slow modifications (e.g., work, school, social relationships). A month-by-month analysis could have been less informative because patients could not recognize significant changes from one time point to the subsequent one.

### 2.5. Data Collection and Statistical Analysis

Patients’ information and clinical data were collected in a database, where clinicians were responsible for data entry. The database was organized on Microsoft Excel, and it consisted of seven pages (socio-demographical information + 6 time points with clinical scores). Statistical analyses were carried out on Microsoft Excel, and they were focused on descriptive measures due to the small sample size. The study design was oriented to show real-world experiences, so in the following sections, a lot of raw data will be reported.

### 2.6. Ethical Considerations

Before being enrolled, patients filled out an informed consent form for the analysis and publication of their clinical data during treatment. Acceptance of the pharmacological trial with esketamine was collected on the preliminary visit and reported in the clinical chart. No off-label procedures were performed. All study procedures were conducted in accordance with Good Clinical Practice (GCP) guidelines and the principles of the Declaration of Helsinki. Ethical approval for the study was granted by the Pavia Ethics Committee during its session on 27 August 2021 (Opinion No. 84157/21), with a subsequent amendment issued under No. 0102231/21.

## 3. Results

### 3.1. Patient 1

G. is a 25-year-old Italian young woman presenting with moderate ASD (Level 2) without intellectual disability (IQ 107). She is currently enrolled at the University of Pavia and works part-time as an assistant for a local writer. Family history is notable for psychiatric disorders (mother: mild-to-moderate depression, father: panic disorder). No neurodevelopmental disorder was diagnosed during childhood. The first depressive episode occurred in 2019 after enrolling in university. She was hospitalized in the Psychiatric Department of Pavia due to the development of SI. During hospitalization, symptoms of hyperactivity and racing thoughts were observed, resulting in an initial diagnosis of bipolar disorder. She was discharged with a prescription for aripiprazole and lithium, but depressive symptoms and SI recurred. Subsequent treatments with vortioxetine and lurasidone yielded no significant improvement. Rigid thinking, hyper-focus on restricted/repetitive activities, and difficulty in recognizing emotions led clinicians to the suspicion of autism, which was confirmed. She began fluoxetine, later switched to sertraline (200 mg/day). Additionally, she participated in weekly group psychotherapy, which offered further support. In early 2021, due to the persistence of depressive symptoms and self-harming behaviors (cutting and cigarette burns), a trial with esketamine was carried out. After six months of treatment, the patient requested to stop the administration. After about a year of stable functioning in both social and occupational domains, a new depressive episode occurred. She resumed treatment with esketamine in January 2023, and she maintained treatment for six months, during which the follow-up protocol was carried out. She received the last administration of the drug in June 2023. Since then, G. has been monitored through periodic outpatient evaluations. In 2024, she discontinued group psychotherapy, maintaining psychiatric follow-up. At the end of 2025 clinical picture was substantially Thanstable.

**Figure 1:**
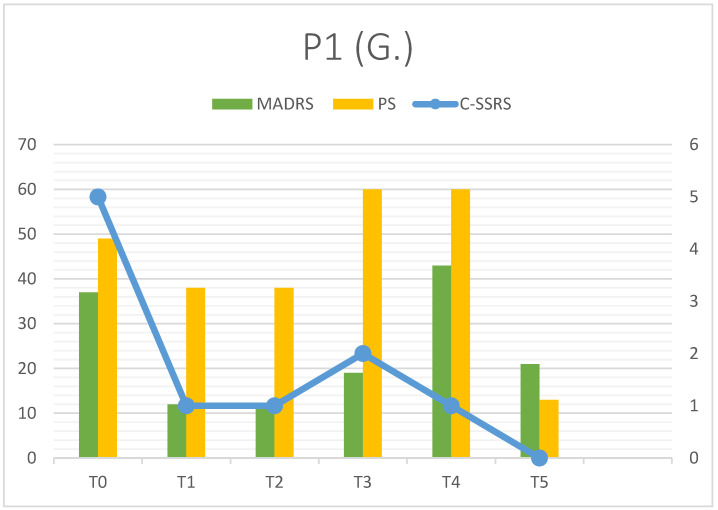
P1 outcomes (depressive symptoms).

G. showed a response to esketamine, with an overall reduction in depressive symptoms from T0 (MADRS = 37) to T5 (MADRS = 21) (Figure 1, Table 3). A minor side effect was reported (one episode of nausea with vomiting, T4) without serious consequences for the patient. A major worsening during treatment (T3–T4) was observed in response to a traumatic trigger (interpersonal trauma). Even if clinical response was partial (dMADRS T0–T5 = 43.24%), clinical intercourse must be considered. Before the traumatic event, G. showed a solid response maintained for more than a month (MADRS = 12 at T1, confirmed at T2, dMADRS = 67.57%). SI was present at T0 with high intensity (5/5) and was totally suppressed at T5 despite the depressive relapse, when there was a minor restart (2/5 at T3). Psychache maintained high values throughout the observation period, decreasing only at T5 (13): interestingly, high values were reached after trauma (T3 = 60, T4 = 60). Mentalization was underperforming at T0, but RFQ-8 reached its highest score (=greater lack of mentalization) at T3, when G. reported a self-harming episode (cutting). The RMET score 31.33 ± 2.66 showed good social cognition, which seemed to improve during therapy. Q-LES-Q reported a significant improvement in quality of life (T0 = 28%, T5 = 73%), particularly in leisure time (T0 = 32%, T5 = 80%).

### 3.2. Patient 2

E. is a 20-year-old Italian male diagnosed with Level 2 ASD with a high cognitive profile (IQ 137). He graduated from high school in 2023 with a qualification as a surveyor, and now he is employed on a farm through a rehabilitation program coordinated by the mental health services of Pavia. His family history is positive for psychiatric conditions (mother diagnosed with bipolar disorder). E. first accessed psychiatric services in the summer of 2023 for a serious self-harming episode (he intentionally set himself on fire at home). The self-destructive act resulted in extensive third-degree burns to his face, trunk, upper limbs, abdomen, and proximal lower limbs. He underwent multiple homografts, resulting in a long hospitalization, which was complicated by sepsis (caused by ESBL-producing *S. aureus* and *A. baumannii*) and bilateral femoral deep vein thrombosis.

Upon the transfer to the Psychiatric Ward of Pavia, clinical observations suggested high-functioning autism characterized by rigid thought patterns, restricted interests, limited social network, hyper-academic performance, reduced emotional sharing, resistance to change, controlling behaviors, and childhood-onset motor tics. The severe self-harming event was described with emotional detachment and attributed to impulsivity triggered by stressful life events. Antidepressant therapy with sertraline resulted in sub-optimal results and was subsequently replaced with venlafaxine (300 mg/day), leading to improvement and discharge. Aripiprazole was also prescribed, with a good effect in mood stabilization. Depressive relapse occurred in late 2023, and he was re-hospitalized for SI; esketamine was introduced during his inpatient stay (February 2024). The administration followed a consultation with an internist to manage hypertension related to severe obesity and metabolic syndrome (propranolol was prescribed). After the first 14 days of treatment, the patient was discharged and continued esketamine as an outpatient: therapy with esketamine is still ongoing, but our follow-up ended in August 2024. Besides esketamine, he is receiving group psychotherapy and educational support. Nowadays, E. is maintaining a quite good functioning considering job and relationships. No more suicidal behaviors occurred. In 2025, aripiprazole was switched to lurasidone due to some gambling episodes reported by E. to clinicians.

**Figure 2:**
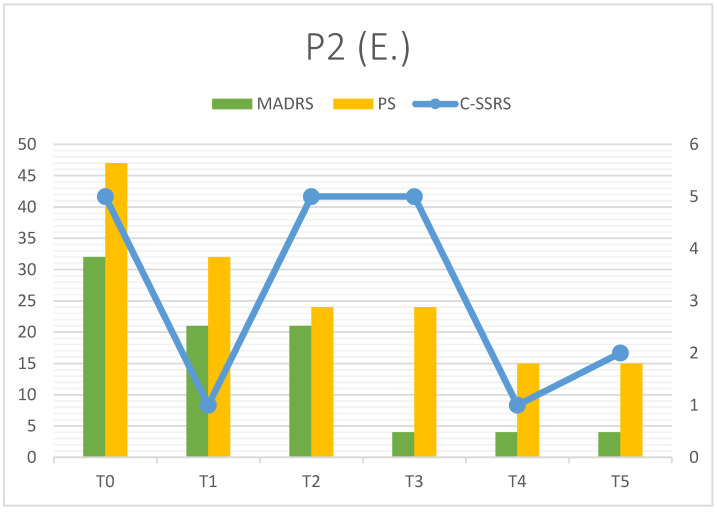
P2 outcomes (depressive symptoms).

Also, E. showed a good clinical response to esketamine with a constant decrease in MADRS and PS scores from T0 to T5 (Figure 2; Table 4). Clinical remission at T5 was achieved (MADRS = 4), but SI showed a complex trend: it was elevated at T0 (intensity 5/5), after a significant improvement at T1 (1/5), it maintained high values for two months, decreasing only in the terminal part of the follow-up (at T5: 2/5, mild). SI did not determine other suicidal or para-suicidal behaviors, and this may be associated with a good ability of mentalization (RFQ-8 2.125 ± 0.209), which apparently improved during therapy. Discharge from the hospital occurred a little before T2, and in our opinion, this could explain the SI restart (patient returned home, where he organized the suicidal attempt). Psychache showed a solid, constant decrease (47 at T0, 24 at T2–T3, 15 at T5). On the other hand, RMET 23.33 ± 1.36 underlined significant impairment in social cognition, as described in the neurodivergent population (see before).

E. perceived an improvement in quality of life (T0 = 25%, T5 = 71%), where the main part was represented by the work domain: he was unemployed at T0, at T5 the rehabilitative program was just started, and the patient reported 92% of functioning.

### 3.3. Patient 3

A. is a 25-year-old woman born in Romania, but who has lived in the United Kingdom, where she graduated in neurosciences at the University of Cardiff. After graduation, she moved to Milan to pursue a PhD in neuropsychopharmacology. During childhood, she faced early scholastic difficulties. Intellectual disability and specific learning disorders were excluded, so a hypothesis of a neurodevelopmental disorder was made. A diagnosis of ADHD was carried out considering the profile of hyperactivity and inattention, which are responsible for most difficulties at school. Methylphenidate was prescribed but was early interrupted due to poor compliance. Nevertheless, her academic path was completed without rejection. At the age of 22, the diagnosis of ASD Level 1 was performed in consideration of persistent social avoidance, rigid thinking, and/or behavioral patterns and hypersensitivity. IQ was evaluated, and good cognitive functioning was confirmed (130). The first MDD occurred in 2018 during high school; after remission, she underwent three depressive episodes not requiring hospitalization. No evidence of family psychopathology. Many pharmacological approaches were used: several SSRIs were prescribed without significant effect, SNRIs were poorly tolerated (different and quite unclear side effects), and a tricyclic antidepressant (amitriptyline) determined only a partial response. Augmentation strategies with mood stabilizers (lamotrigine) and second-generation antipsychotics (risperidone, aripiprazole, quetiapine, amisulpride) were useless. Methylphenidate was prescribed again in Italy (50 mg/day), and it offered a partial improvement, considering cognitive symptoms. In accordance with the patient. Esketamine was prescribed in 2025, adding on oral therapy with escitalopram up to 20 mg/day; this therapy is still ongoing.

**Figure 3:**
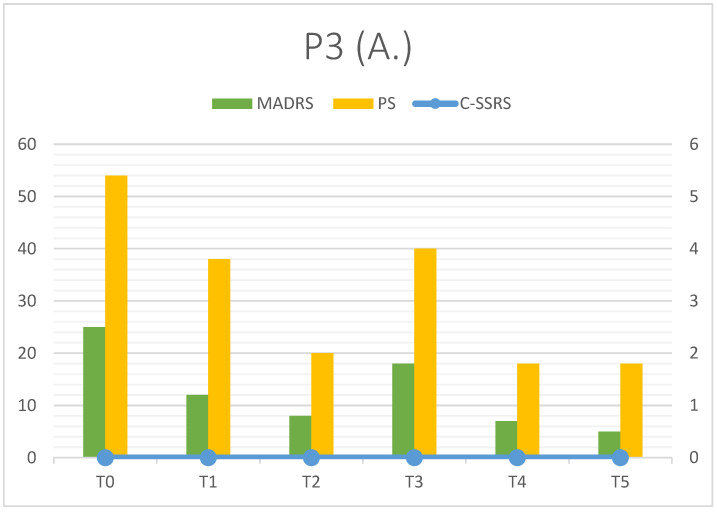
P3 outcomes (depressive symptoms).

With esketamine, A. underwent a significant improvement, as testified by a rapid decrease in MADRS and PS scores (Figure 3; Table 5). Also, A. reached clinical remission at T5 (MADRS = 5) It is possible to notice an isolated peak in the MADRS score (18) at T3, when A. confessed that she had suspended methylphenidate for a second time. This led to a rapid worsening in cognitive symptoms detected with MADRS. This also showed the importance of specific treatment in ADHD comorbidity; despite some resistance, A. demonstrated good compliance to therapy, resuming the pharmacotherapy with methylphenidate soon after the visit. SI was absent from the beginning, and C-SSRS = 0 remained stable during follow-up. Psychache also saw a solid improvement (54 at T0, 18 at T5) and, as MADRS, it showed a relative peak at T3 (40). Mentalization was sufficient at T0 (2), but it was characterized by an apparent worsening during follow-up (T5 = 4) in the absence of dysregulation episodes. RMET score 26.83 ± 1.47 was comparable to the neurotypical mean and showed a little increase in six months.

A. reported an increased quality of life at T5 (80% vs. 35% at T0), the bigger improvement was noted in social relations (percentage of satisfaction moved from 30% at T0 to 84% at T5). 

## 4. Discussion

### 4.1. Summary of Findings and Interpretation

Preliminary data is not enough to formalize strong conclusions, so this small case series can be considered as exploratory work; moreover, the case series design does not permit statistical inferences. In our experience, esketamine seemed to be efficacious in patients with TRD and ASD, with a good safety profile: all patients tolerated the pharmacological intervention well, in the absence of severe side effects. Nausea with vomiting was noted in one episode (P1, T4), confirming the profile of minor side effects known for esketamine. Vital signs monitored during administrations (blood pressure, temperature, heart rate, blood oxygen saturation) showed no significant modifications in six months (see Supplementary Material).

All patients observed showed a rapid decrease in MADRS total score from T0 to T1 (first week). P1 and P3 reached a significant clinical response (dMADRS of 67.57% and 52% respectively), while P2 showed a partial response in the first week (dMADRS = 34.38%). Considering the whole observation period (T0–T5), P2 and P3 achieved the primary outcome, with clinical remission in depressive symptoms (MADRS < 10). P1’s response was partial due to a depressive relapse that occurred during treatment. Esketamine seemed to improve other symptoms investigated as secondary outcomes: psychache decreased in all patients, such as suicidal ideation, which could be the most dangerous symptom in depression with autism comorbidity. It is interesting that suicidal thoughts, when present, are partially linked to depressive trend, but they can follow a quite separate trajectory (Figure 1 and Figure 2). In our opinion, this finding should point out the importance of a specific diagnosis and follow-up on suicidal ideation to prevent serious consequences for patients; in particular, patients with TRD and ASD may be associated with an elevated risk of suicide. Esketamine add-on showed promising results in decreasing suicidal ideation, and this could be related to the specific pharmacological profile (NMDA receptor blocking with sedation and mild dissociative effect). Administration setting (repeatedly accessing mental health services, multiple contacts with clinicians and/or nurses) could also assume a significant role in suicidality prevention. Mentalization and social cognition did not show substantial modification during treatment, but data testified to the clinical heterogeneity of these features in people affected with ASD, suggesting the importance of personalized approaches. Furthermore, subjective quality of life was characterized by a constant increase in six months; this could suggest the efficacy of Esketamine in reducing the loss of function observed in depressive episodes, but also the good tolerance to the therapy setting (frequent access, clinical monitoring). As our patients reported in follow-up visits, Esketamine administrations were not associated with organizational problems and/or modifications in everyday life.

The last consideration is about clinical stressors impact; we tried to explain the reason why these three patients with similar diagnoses showed different responses to esketamine. This pharmacological intervention was substantially effective in all patients, as the rapid decrease in depressive symptoms testified (see dMADRS T0–T1). Confirmation of clinical response during the observation period led to remission at T5 (P2 and P3), whereas P1 improvement was limited by an external factor (trauma), which caused a major relapse. Trauma was limited to a specific episode just before T3 (MADRS = 19), but depressive symptoms were more pronounced at T4 (MADRS = 43). After this point, another improvement was observed with a strong decrease at T5 (MADRS = 21) in the absence of clinical remission. Trauma represented a worsening factor, but, in our opinion, mentalization also played a key role. The theory of mind implies understanding social situations but also facts and/or behaviors, allowing a better tolerance of everyday life RFQ-8 and RMET scores can be considered as exploratory signals, but their values during follow-up could assume clinical significance. P1 had been associated with the higher RFQ-8 mean score (3.83 ± 0.71), and after trauma, she showed a major relapse; P2 showed quite good mentalization ability during the observation period (RFQ-8 = 2.13 ± 0.21), and this could have represented a protective factor from active SI (T2–T3), preventing suicidal behaviors observed before treatment. P3 also scored high RFQ-8 (3.33 ± 0.82), but in the absence of precipitating causes, worsening did not occur; it is important to notice that her anamnesis at baseline was negative for both suicidal ideation and behaviors.

### 4.2. Comparison to Literature

Our research work was focused on the efficacy and safety of intranasal esketamine in a special population (people with TRD and comorbid ASD). Autism is a frequent comorbidity among psychiatric disorders, and there are a lot of autistic patients who suffer from depressive episodes. Nevertheless, there are no studies that investigate the therapeutic role of esketamine in people with ASD. MADRS score was selected to monitor depressive symptoms during follow-up, as in most of the studies that investigated esketamine efficacy. Obviously, our study could not be associated with statistical analysis, so we adopted a descriptive approach. Reported results indicate a similar response to esketamine at six-month follow-up [23]. An early decrease in MADRS score was noted during the induction phase (first month, two administrations/week), and it was confirmed during the maintenance phase (second month, one administration/week; months 3 to 6, personalized frequency). The clinical trend seemed comparable to other studies for both timing and efficacy; in particular, a solid improvement during the first month of treatment was also noted in our patients with ASD as a comorbidity [24]. Considering the safety profile, adverse events reported by our patients were very mild, and they included one nausea episode with vomiting (P1). Headache and major increases in blood pressure were never noted; mild dizziness was often described, but it was reported as a quite pleasant event.

### 4.3. Strengths and Limitations

In our opinion, the strong points of these case reports are the focus on an understudied population (people who suffer from ASD and TRD) and the innovative approach with esketamine + SSRI/SNRI in young patients who have undergone few pharmacological trials. Considering autism as a condition associated with severe depression and treatment resistance led us to prescribe esketamine add-on rather than proceeding with other antidepressant strategies: clinical outcomes seemed to enforce this choice. The presentation of raw data (Table 3, Table 4 and Table 5) could be useful to draw a real-world experience, and it may also constitute a term of comparison for further studies.

The main weakness of the research is the elevated inter-individual variability with no standardization (difference in oral antidepressant therapy, duration of esketamine trial, and administration frequency). Other limitations are the lack of placebo controls, the lack of a control group (neurotypical people treated with esketamine for TRD), the short follow-up period, and the absence of blinding (assessor bias). Publication bias is not mentioned because patients recruited for the study were the only TRD patients with a co-diagnosis of ASD treated in our clinics (2025).

### 4.4. Future Research Directions

We hope that this case series could be the basis for larger studies. The first step could be an observational study with a bigger sample to confirm the preliminary results of efficacy and tolerance. Then, a case–control analysis could be set up; autistic patients treated with TRD treated with esketamine could be compared to neurotypical patients, checking possible differences in terms of clinical response. Also, blind or double-blind studies would be easier to set up, investigating the effective action of esketamine compared to placebo or other antidepressants. Finally, it will be interesting to consider other variables, such as neuro-functional biomarkers (fMRI and/or EEG correlates).

## 5. Conclusions

In our experience, esketamine represented a valid treatment for TRD in patients with ASD. It was characterized by good efficacy, with a rapid and significant decrease in depressive symptoms, including suicidal ideation, when present. The pharmacological profile was favorable in terms of safety and tolerability; no major side effects were reported. With more studies and scientific evidence, esketamine could become a therapeutic option for patients with MDD and comorbid autism, who were often characterized by resistance to treatment and moderate to high suicidal risk.

## Figures and Tables

**Table 1:** Clinical and demographical features.

	PATIENT 1 (G.)	PATIENT 2 (E.)	PATIENT 3 (A.)
GENDER AND AGE	Female, 25 y	Male, 20 y	Female, 25 y
JOB	Student Writer assistant	Farm worker	Student
AUTISM SEVERITY	Level 2	Level 2	Level 1
INTELLECTUAL DISABILITY	No (IQ 107)	No (IQ 137)	No (IQ 130)
FAMILIAR ANAMNESIS	Major depressive disorder (mother) Panic disorder (father)	Bipolar Disorder (mother)	Negative
MEDICAL COMORBIDITIES	No	Sever obesity, hypertension	No
HOSPITALIZATIONS	Suicidal ideation (2021)	Suicidal attempt (2023) Suicidal ideation (2024)	No
SUICIDAL ATTEMPTS	No	1 (setting fire)	No
SELF-HARMING	Yes (cutting, cigarette burns)	No	No
DRUG USE	No	Alcohol (occasional)	No
ORAL PHARMACOTHERAPY	Sertraline 200 mg/day Aripiprazole 15 mg/day	Venlafaxine 300 mg/day Lurasidone 74 mg/day	Escitalopram 20 mg/day Methylphenidate 50 mg/day
ESKETAMINE POSOLOGY	84 mg	84 mg	84 mg
ESKETAMINE DURATION	6 months	15 months (ongoing)	9 months (ongoing)
HOSPITALIZATIONS DURING ESKETAMINE	No	No	No
SUICIDAL ATTEMPTS DURING ESKETAMINE	No	No	No
SELF-HARMING DURING ESKETAMINE	Yes (1 episode)	No	No

**Table 3:** P1 results.

P1	MADRS	C-SSRS	PS	RFQ-8	RMET	Q-LES-Q (%)
**T0**	37	5	49	4.125	27	28
**T1**	12	1	38	4.25	31	-
**T2**	12	1	38	4.25	30	-
**T3**	19	2	60	4.5	32	-
**T4**	43	1	60	3.125	34	-
**T5**	21	0	13	2.75	34	73

**Table 4:** P2 results.

P2	MADRS	C-SSRS	PS	RFQ-8	RMET	Q-LES-Q (%)
**T0**	32	5	47	2.5	26	25
**T1**	21	1	32	2.25	22	-
**T2**	21	5	24	2	23	-
**T3**	4	5	24	2	23	-
**T4**	4	1	15	2	23	-
**T5**	4	2	15	2	23	71

**Table 5:** P3 results.

P3	MADRS	C-SSRS	PS	RFQ-8	RMET	Q-LES-Q (%)
**T0**	25	0	54	2	26	35
**T1**	12	0	38	3	27	-
**T2**	8	0	20	4	25	-
**T3**	18	0	40	3	26	-
**T4**	7	0	18	4	28	-
**T5**	5	0	18	4	29	80

## Data Availability

This case series consists of clinical and demographic information. Clinical data are in large part raw data due to the preliminary and descriptive nature of the research. The dataset has a dedicated section for each patient, and it will be available on request from the authors. Also, demographic data is available on request due to ethical restrictions (privacy).

## Supplementary Materials

The following supporting information can be downloaded at: https://www.mdpi.com/article/10.3390/clinpract16030061/s1. Figure S1: Treatment outline (P1).; Figure S2: Treatment outline (P2).; Figure S3: Treatment outline (P3).
