# Supplementary material for: Efficacy and Safety of Intranasal Esketamine in Treatment-Resistant Depression with Comorbid Autism Spectrum Disorder: Three Case Reports
Source: Clin Pract. 2026 Mar 13;16(3):61. doi: 10.3390/clinpract16030061 (PMC13024768; doi:10.3390/clinpract16030061)
Supplement: Supplementary file 1 [file clinpract-16-00061-s001.zip › clinpract-4118402-supplementary.pdf]

Supplementary Material

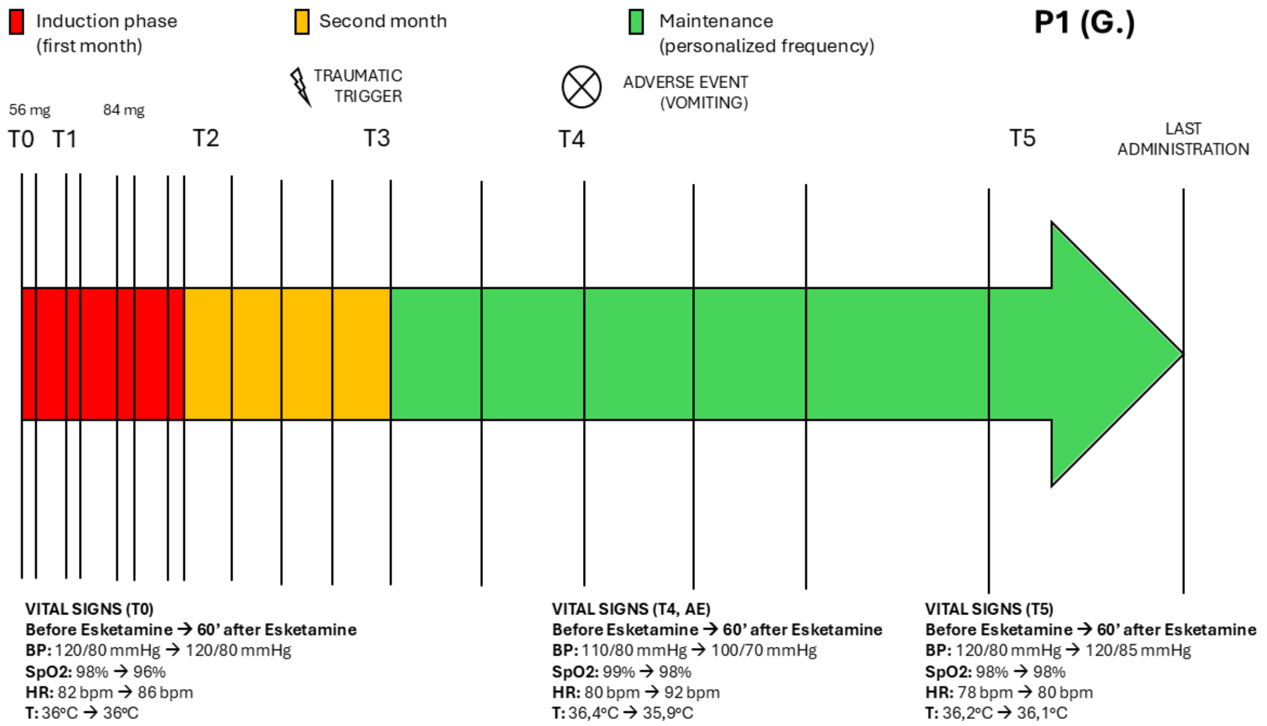

Figure S1. Treatment outline (P1).

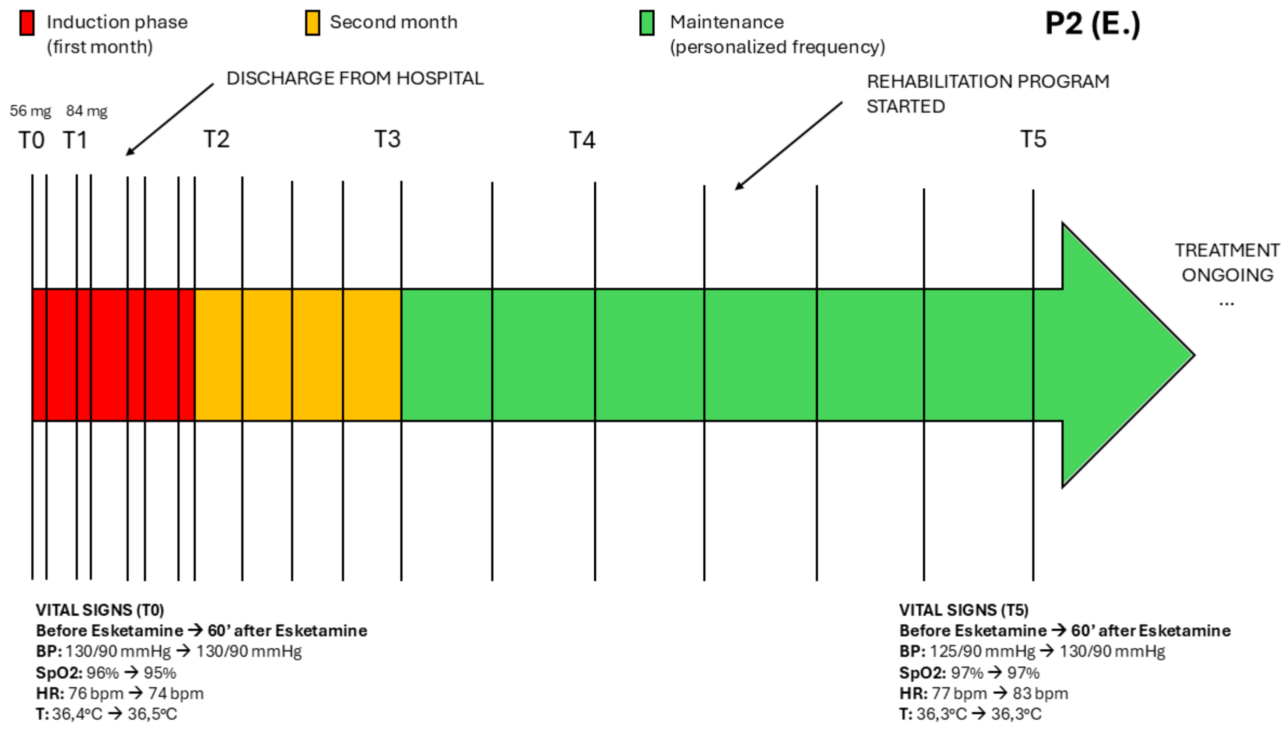

Figure S2. Treatment outline (P2).

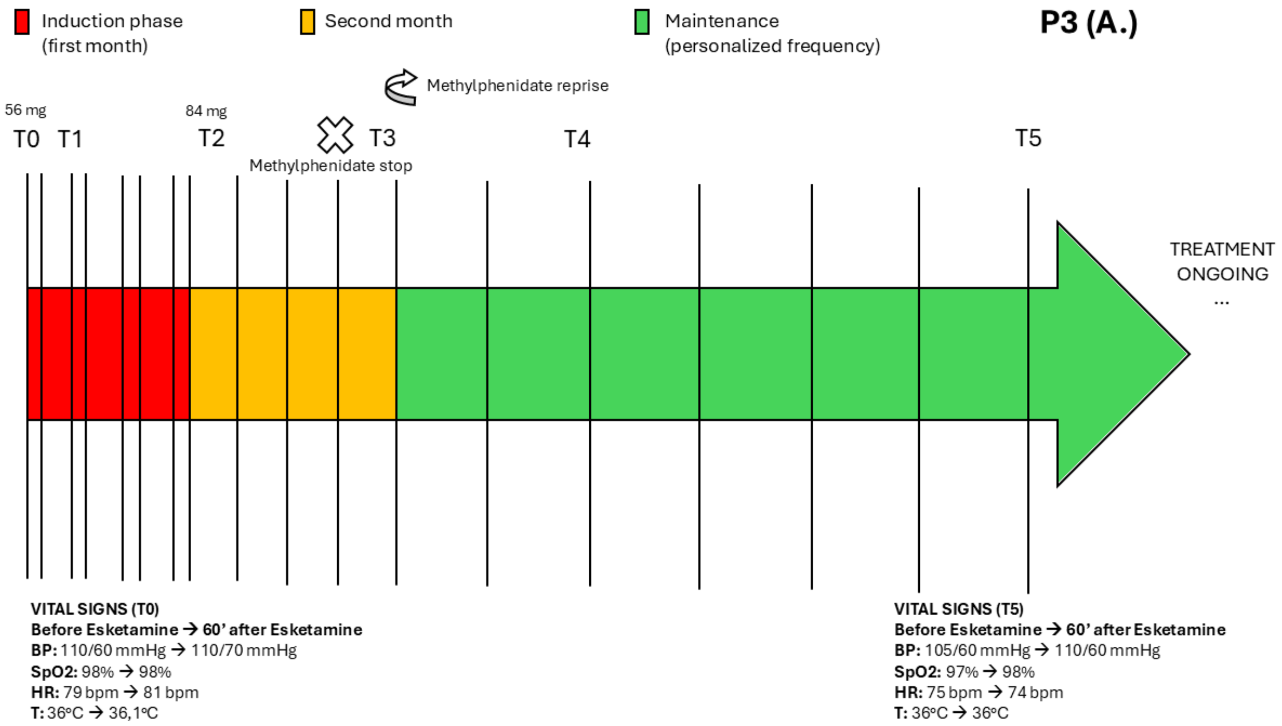

Figure S3. Treatment outline (P3).
